# First report on aberrant *Ascaris suum* infection in a dog, China

**DOI:** 10.1186/s13071-020-3963-0

**Published:** 2020-02-18

**Authors:** Yue Xie, Yunjian Liu, Xiaobin Gu, Xuan Zhou, Xuerong Peng, Ran He, Hongrui Guo, Zhicai Zuo, Guangyou Yang

**Affiliations:** 1 Department of Parasitology, College of Veterinary Medicine, Sichuan Agricultural University, Chengdu, 611130 China; 2 Institute of Animal Genetics and Breeding, College of Animal Science and Technology, Sichuan Agricultural University, Chengdu, 611130 China; 3 Department of Chemistry, College of Life and Basic Science, Sichuan Agricultural University, Chengdu, 611130 China; 4 Key Laboratory of Animal Diseases and Environmental Hazards of Sichuan Province, Sichuan Agriculture University, Wenjiang, Chengdu, 611130 China; 5 Key Laboratory of Animal Disease and Human Health of Sichuan Province, College of Veterinary Medicine, Sichuan Agricultural University, Chengdu, 611130 China

**Keywords:** Ascariasis, *Ascaris suum*, Helminthic zoonosis, Host range, Dogs

## Abstract

An aberrant *Ascaris suum* infection in a domestic dog in China in 2019 is described for the first time. This pathogen is a common roundworm of pigs with few reported cases in domestic animals. Our findings suggest a wider infection range with a possible transmission of *A. suum* to domestic animals that interact with humans.

**Letter to the Editor**

Roundworms, belonging to the family Ascarididae, are among the most common zooparasitic nematodes and cause ascariasis in all major lineages of vertebrates including domestic animals, wildlife and humans. *Ascaris suum* is a common roundworm of pigs. Although pig husbandry has become more modernized and industrialized, *A. suum* is still prevalent on a large number of pig farms across the world and the situation has not changed significantly over the last few decades [1]. It is evident that *A. suum* is zoonotic and its cross-infection and hybridization with the human roundworm *Ascaris lumbricoides* has been recently confirmed in areas of human-pig sympatry [2]. Importantly, cases of *A. suum*-related human infections have been reported [3–5], including an outbreak in Maine, USA, in 2010–2013 [6]. In addition, aberrant *A. suum* infections were also reported in domestic animals, such as cattle [7]. Here we expand the currently recognized infection spectrum by firstly describing *A. suum* infection in domestic dogs in China.

In April 2019, a two-month-old female German shepherd dog puppy from a pig farm was brought to Veterinary Medical Teaching Hospital (VMTH), Sichuan Agricultural University (Sichuan, China), for *post-mortem* examination. The puppy died with clinical manifestations of dyspnea, coughing, wheezing and general weakness. This farm kept five German shepherd dogs (a bitch and her four puppies), and all the puppies had shown similar clinical signs for about two weeks. Routine vaccinations were administrated to all of the dogs.

Necropsy of the puppy revealed bilateral lungs with significant emphysema and diffusely firm red parenchyma, and the trachea and bronchi were filled with massive blood-tinged froth. Interstitial pneumonia was diagnosed according to the gross appearance, and viral or bacterial pneumonia was suspected. However, histopathological examination showed a diffuse, severe, hemorrhagic, fibrinous interstitial pneumonia with multiple nematode larvae present in alveolar sacs and bronchi which were partially surrounded by inflammatory cells (macrophages, lymphocytes and eosinophils) (Fig. 1a, b). The larvae measured 68 μm in maximum transverse diameter and had a thin cuticle that formed sharply pointed paired lateral alae. The intestine was centrally located, floated in pseudocoelom and flanked by large paired triangular excretory columns. Four to five muscle cells were observed per quadrant. These morphological characteristics are key for ascaridid nematodes. Examination of gastrointestinal tracts from the puppy recovered three acaridid nematodes. No viral or bacterial etiology was diagnosed from routine bacteriological and molecular diagnostic tests.

**Fig. 1:**
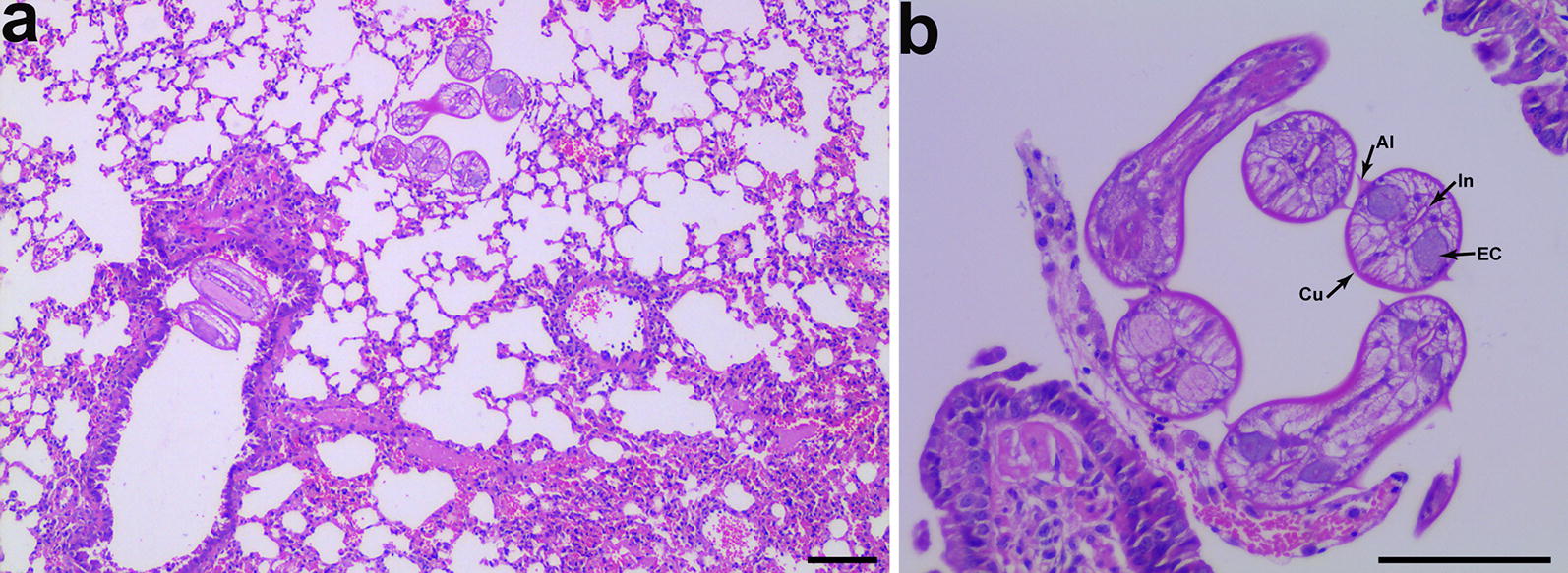
Morphological characterization of *Ascaris suum* nematode infection in the lung of a dog in China, 2019. **a** Multiple transverse sections of *A. suum* larvae observed in lung tissues with severe, hemorrhagic, fibrinous interstitial pneumonia. **b** The morphological characteristics of the larvae included maximum diameter of 68 μm, thin cuticle (Cu), a centrally located intestine (In), paired lateral alae (Al) and excretory columns (EC). Some scattered macrophages, lymphocytes and eosinophils are also indicated. Hematoxylin and eosin stain. *Scale-bars*: 100 µm

The three acaridid nematodes were provisionally identified as *Toxocara canis* based on morphology: the presence of a post-oesophageal bulbus, the length and shape of the alae and the length of the spicules. *Toxocara canis* is a canine roundworm that is highly prevalent in puppies under three months-old and can adopt “hepato-tracheal” migration to develop into adults. Thus, this parasite can also cause histopathological changes of lungs similar to those identified in this study.

To accurately identify the nematodes in the infected lung, genomic DNA was extracted from formalin-fixed, paraffin-embedded (FFPE) tissues and subjected to genetic analysis by sequencing partial fragments of the nuclear *18S* ribosomal DNA (*18S* rDNA) gene [8] and the mitochondrial *12S* ribosomal DNA (*12S* rDNA) gene [9]. The resulting sequences of *18S* rDNA (1729 bp; GenBank: MN558962) and *12S* rDNA (488 bp; GenBank: MN567666) were found to be 99.9–100% identical with those of *A. suum* (GenBank: U94367 and FJ418791, respectively), and both grouped together and were clearly distinct from *T. canis* in phylogenies inferred from either *18S* rDNA or *12S* rDNA using maximum likelihood and Bayesian inference methods (Fig. 2a, b). Indeed, further investigations supported pigs as the infection source because *c.*60% pigs in this farm were serologically detected as *A. suum*-positive by AsHb ELISA (Y. Zheng et al., unpublished data) [10], and parasitological examination of soil and water around the pig farm indicated a heavy contamination with *A. suum* eggs. This suggested that the free-range German shepherd dogs were probably exposed to the eggs and became infected.

**Fig. 2:**
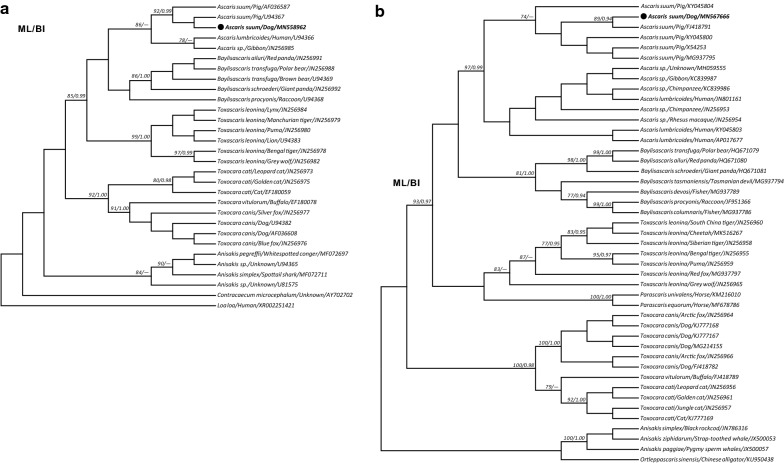
Molecular characterization of *Ascaris suum* nematode infection in the lung of a dog in China, 2019. Phylogenetic trees inferred by using Tamura-Nei model-based maximum likelihood (ML) and GTR+I+G model-based Bayesian inference (BI) analyses based on the partial sequences of the nuclear *18S* rDNA gene (**a**) and the mitochondrial *12S* rDNA gene (**b**) of the isolate from the dog (bold and marked with a solid black circle) and other related species. *Loa loa* (**a**) and species of *Anisakis* and *Ortleppascaris* (**b**) were used as outgroups. The numbers along the branches indicate bootstrap values resulting from different analyses in the order ML/BI; values > 70% (ML) and 0.95 (BI) are shown

The pig roundworm *A. suum* has been suggested as causing a widely distributed zoonosis with human cases in North America, Europe and Asia [2, 3, 6]. Some other animals infected with *A. suum* have been also recently reported [7]. Our finding shows that this roundworm can infect dogs. This observation expands the previously acknowledged infection range and raises questions about infection success and host affiliation of *A. suum* in nature that are difficult to address by traditional biological, epidemiological and clinical approaches. Moreover, in consideration of the cultural and agricultural tradition of using pig feces as fertilizer and the feature that *A. suum* eggs remain viable in farm soil for years as well as the location of pig latrines where dogs easily access, the chance of direct or indirect exposure for dogs is likely. Recommended preventive procedures to reduce transmission of *A. suum* between pigs and dogs, and probably between pigs and humans, include restraining dogs from pig lactrines, periodically examining farm dogs and pigs for infection, prophylactic and therapeutic treatment of pigs to reduce infection, avoiding use of pig manure as fertilizer, and washing hands when there is contact with pigs, pig waste or soil contaminated with pig waste. In addition, the farm owner should have dedicated equipment for handling or depositing animal waste. Because *A. suum* eggs can persist in the farm surrounding for extended periods, daily foodstuffs, especially produce and vegetables grown using pig manure should be avoided or fed only after thorough cleaning. Overall, an effective prevention of *A. suum* infections requires an integrated “One-Health” approach that encourages collaboration between farmers, veterinarians and physicians and enhances their awareness to the risk for aberrant infections of *A. suum* and its transmission to humans and other domestic animals.

## Data Availability

Nucleotide sequences reported in this article are available in the GenBank database under the accession numbers MN558962 (*18S*) and MN567666 (*12S*).
